# Interventions for improving management of chronic non-communicable diseases in Dikgale, a rural area in Limpopo Province, South Africa

**DOI:** 10.1186/s12913-018-3085-y

**Published:** 2018-05-04

**Authors:** Eric Maimela, Marianne Alberts, Hilde Bastiaens, Jesicca Fraeyman, Herman Meulemans, Johan Wens, Jeane Pierre Van Geertruyden

**Affiliations:** 1 Department of Pathology and Medical Sciences, School of Health Care Sciences, University of Limpopo, Turfloop campus, P O Box 1043, Duiwelskloof, 0835 South Africa; 2 Department of Pathology and Medical Sciences, School of Health Care Services, University of Limpopo, Turfloop campus, Private Bag X1106, Sovenga, 0727 South Africa; 3 Department of Primary and Interdisciplinary care, University of Antwerp, Universiteitsplein 1, 2600 Antwerp, Belgium; 4 Research group Medical Sociology and Health Policy, University of Antwerp, Universiteitsplein 1, 2600 Antwerp, Belgium; 5 Department of Sociology and Research Centre for Longitudinal and Life Course Studies, University of Antwerp, Sint-Jacobstraat 2, BE-2000 Antwerpen, Belgium; 6 Centre for Health Systems Research & Development, University of the Free State, Bloemfontein, South Africa; 7 Department of Primary and Interdisciplinary care, University of Antwerp, Universiteitsplein 1, 2600 Antwerp, Belgium; 8 International Health Unit, University of Antwerp, Universiteitsplein 1, 2600 Antwerp, Belgium

**Keywords:** Community health services, Health care reform, Health planning, Interdisciplinary communication, Managed care programs, Primary prevention

## Abstract

**Background:**

Chronic disease management (CDM) is an approach to health care that keeps people as healthy as possible through the prevention, early detection and management of chronic diseases. The aim of this study was to develop interventions to improve management of chronic diseases in the form of an integrated, evidence-based chronic disease management model in Dikgale, a rural area of Limpopo Province in South Africa.

**Methods:**

A multifaceted intervention, called ‘quality circles’ (QCs) was developed to improve the quality and the management of chronic diseases in the Dikgale Health and Demographic Surveillance System (HDSS). These QCs used the findings from previous studies which formed part of the larger project in the study area, namely, the quantitative study using STEPwise survey and qualitative studies using focus group discussions and semi-structured interviews.

**Results:**

The findings from previous studies in Dikgale HDSS revealed that an epidemiological transition is occurring. Again, the most widely reported barriers from previous studies in this rural area were: lack of knowledge of NCDs; shortages of medication and shortages of nurses in the clinics, which results in patients having long waiting-time at clinics. Lack of training of health care providers on the management of chronic diseases and the lack of supervision by the district and provincial health managers, together with poor dissemination of guidelines, were contributing factors to the lack of knowledge of non-communicable diseases (NCDs) management among nurses and community health care workers (CHWs). Consideration of all of these findings led to the development of model which focuses on integrating nursing services, CHWs and traditional health practitioners (THPs), including a well-established clinical information system for health care providers. A novel aspect of the model is the inclusion of community ambassadors who are on treatment for NCDs and are, thus, repositories of knowledge who can serve as a bridge between health care workers and community members.

**Conclusion:**

The model developed highlights the need for health interventions that aim to control risk factors at the population level, the need for availability of NCD-trained nurses, functional equipment and medication and a need to improve the link with traditional healers.

## Background

Chronic health conditions represent a substantial challenge to global health and by 2020 they will account for 73% of all deaths, constituting 60% of the global burden of disease [1]. In South Africa NCDs have increased over the past 15 years. They now account for an estimated 37% of all-cause mortality and 16% of disability-adjusted life years [2]. The overall level of NCD mortality is similar across all provinces in South Africa but the causes are different [3]. Most NCDs share risk factors [4], and many of them are modifiable [5] and are usually adopted early in life [3]. This provides considerable opportunities for intervention [2, 5]; however, progress in reducing NCD risk factors will only be attained if appropriate attention is given to the social and cultural contributing factors [2].

Chronic disease management (CDM) entails an integrated approach involving the patient, the family and the community as active participants over a lifetime of care [6, 7]. Effective interventions to limit the progression of the diseases or to mitigate the risk of complications are needed [8]. Most CDM interventions should be tailored to the person’s strengths and challenges in managing their care [9]. Thus, collaborative efforts on the management of people with chronic diseases in primary health care (PHC) settings plays an important role in CDM, as PHC professionals link their services to other specialised services in the communities [10]. Therefore, PHC requires a greater level of organisation that must be sustained, commonly over a patient’s lifetime [11]. Developing these interventions is a challenge in low-income and middle-income countries [11]. The aim of this study was to develop an integrated, evidence-based CDM model based on the premise that multiple-strategy interventions are consistently more effective than single-strategy interventions [12].

## Methods

The research methodology for the current study was guided by the research question, which was developed in order to understand the burden of chronic disease risk factors; and to learn about the perceptions, experiences, barriers and challenges of chronic disease with respect to patients, nurses, CHWs and THPs regarding chronic disease management. This, therefore, brought about the idea of employing a mixed methods approach [13, 14] with the aim of integrating quantitative and qualitative data collection and analysis into a single study in order to develop an intervention program in a form of a model designed to improve the management of chronic diseases in a rural area. The use of this mixed-method methodology included the principle of making a decision on the priority or weight given to the quantitative and qualitative data collection and analysis in the study; the sequence of the data collection and analysis; and, the stage/stages in the research process at which the quantitative and qualitative data are connected and the results are integrated [15]. When used in combination, both quantitative and qualitative data yielded a more complete analysis and they complemented each other, bringing about an understanding of the strengths and weaknesses of quantitative and qualitative research methodologies.

Multiple data was collected using different strategies, approaches and methods [16]. The quantitative techniques used included a cross-sectional study design using the World Health Organization (WHO) STEPwise approach to surveillance of NCD risk factors (WHO STEPS) [17]. The qualitative techniques used included focus group discussions (FGDs) [18], semi-structured interviews [19] and QCs [20, 21]. The present study was developmental in nature because the first method was used sequentially to help inform the second and third methods. This was done in the form of sequential triangulation because the model or interventions were developed in phases, with results from all the phases essential for planning the model development.

A multifaceted intervention using QCs [22–24], was developed during the month of February 2014 to improve the quality and the management of chronic diseases in Dikgale HDSS. This intervention comprised small group sessions of expertise to discuss and provide feedback on prevention, management and control of chronic diseases in rural areas. The QCs have become an important method of quality improvement (QI) in primary care [24]. The rationale behind the use of this research methodology was the fact that it involved gathering together small groups of employees doing similar or related work, who voluntary meet to identify, define, analyze and solve work-related problems or issues [25].

### Study setting and sampling

The study was conducted in Dikgale, a rural area which has a HDSS consisting of 15 villages, with poor infrastructure, situated close to one another in the Capricorn District of the Limpopo Province in South Africa [26]. The organisation of health services for chronic diseases in the study area is based on primary health care. These services are not complemented by more specialised and intensive care settings, such as diagnostic labs, specialty care clinics, hospitals and rehabilitation centres [27].

### Participants of quality circles

The QCs comprised of members representing a wide area of expertise with respect to disease control and prevention. Eligibility criteria included people who work as clinic managers in the study area, people with bachelor’s degrees in nursing or associate degrees, people who have conducted extensive research in chronic disease management, an executive manager in Department of Health in Limpopo and a chronic disease manager in the Limpopo Province. An invitation was sent to several institutions employing people with expertise in chronic disease management and at total of 35 participants (as presented in Table 1) were randomly selected from this group to take part in the study.

**Table 1:** Participants in the quality circles

Category	No
Clinic managers from Dikgale HDSS	3
Sub District Manager	1
Chronic disease programme managers from Department of Health	6
Registrars’ from Polokwane/Mankweng hospital complex	5
Staff and students from University of Limpopo Medical Science Department	10
Health Promotion from Limpopo Department of Health	2
Staff member from MRC/Wits-Agincourt Research Unit in Mpumalanga province	1
A representative from the executive management of Department of Health in Limpopo Province	2
A representative from the Dikgale traditional authority	1
Expertise from Antwerp University in Belgium representing the Unit International Health, Department of Primary and Interdisciplinary care, Department of Sociology and Research Methodology.	4
Expertise from University of Umeå, Sweden	1

Patients, nurses, community health workers and traditional health practitioners were excluded in this study because of the use of the QCs methodology [28], which was seen as an innovative approach to addressing quality improvement activities. However, the responses from this group of people elicited in previous studies, which formed part of the major project, were used within the context of CDM improvement in PHC settings. This process involved the identification of, and discussion about, actual problems faced when addressing chronic diseases and possible solutions to these problems, which were raised by patients, nurses, CHWs, THPs and managers in earlier studies in the same study area.

Next, priorities for an integrated community-based chronic disease approach were set and a start was made on an implementation plan.

### Data collection and analysis

This study took the form of a workshop, which was conducted over three days, in order to discuss problems related to the improvement of CDM. Three small groups of approximately 11 participants were formed and several educational/presentational sessions were held to familiarise the participants with the issues of group dynamics, problem-solving, techniques used in QCs, the role of each member of the group and the procedures used in the QCs. These small groups were tasked to discuss current practices and explore evidence-based practices [22] required to improve CDM in the study area. The groups worked through a series of stages, during which participants were encouraged to analyse problems following a sequential process, in order to find possible causes of these problems and then develop solutions. Groups then formally presented project proposals for consideration [29].

Interventions which formed the integrated CDM model were discussed, taking into account the evidence-based and/or grounded theory aspects of each intervention. The basis for the discussion was the findings from previous studies [27], which formed part of the larger project in the study area, i.e. the STEPwise survey, focus group discussions and semi-structured interviews. The STEPwise survey results dealt with the prevalence of risk factors for NCDs. The focus group discussions results dealt with the perceptions and perspectives of chronic patients and health care providers on CDM [27], addressing NCDs through CHWs and THPs. The semi-structures interviews results dealt with the barriers to, and facilitators for, improving the management of chronic NCDs at PHC level by managers of clinics; and the CHWs programme and chronic disease programme at district and provincial level. Model development was also based upon an assessment of the needs, which were inclusive of consultation with key stakeholders and involved a multidisciplinary approach, where applicable, which considered the optimal and equitable utilisation of healthcare resources [30].

## Results

In the previous studies conducted as part of the bigger project in the study area, the quantitative study revealed a high prevalence of behavioural and biomedical risk factors for NCDs [31]. Approximately one in three study participants were found to be hypertensive, starting at a young age of 15 to 24 years. Approximately 90% of the participants were below the WHO recommendations for fruit and vegetable consumption, while more than half had low physical activity levels. A quarter of the participants were overweight and obese, while one in third had high total cholesterol levels. The qualitative studies [27] revealed that lack of knowledge of chronic diseases was predominant among patients, nurses, CHWs and THPs. Training on CDM was also lacking or insufficient and there was poor supervision of health facility operations by the district and provincial managers. The CHWs were not respected by nurses and their remuneration was not regularly received. A poor relationship was found to exist between THPs and clinic nurses, due to lack of formal referral system. This was primarily due to the lack of a formal structure to represent the THPs in government.

The QCs used the abovementioned findings to discuss specific needs in relation to capacities for chronic disease prevention and management in the Dikgale HDSS. The discussions were focused on the ability to integrate determinants of health approach into programme planning in order to address the root causes of chronic disease. Further discussions addressed the integration of health services in order to improve the management and prevention of chronic non-communicable diseases. Therefore, the model development followed three major steps as follows:

- Firstly, to identify inputs (resources), activities, outputs and outcomes in a form of logic framework to improve management of chronic NCDs;
- Secondly, to identify the prerequisites needed to strengthen integrated evidence-based chronic NCDs;
- Lastly, to develop an integrated evidence-based chronic NCD management model.

### Logical framework for improvement of management and prevention of chronic non-communicable diseases

**Fig. 1:**
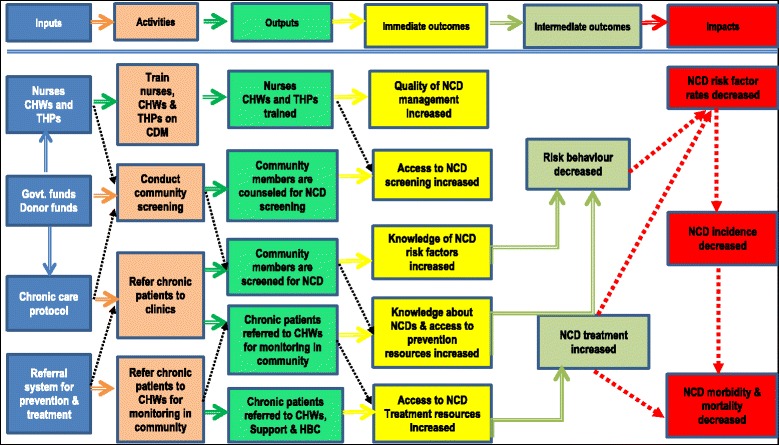
Logic Model to improve management and prevention of chronic non-communicable diseases

A framework in the form of a logic model was established from the results in order to systematically lay out the programme elements and a path showing what could be done to improve management and prevention of chronic NCDs. The logic framework is presented in Fig. 1. The main activities in the framework focus on capacity building for health care providers; community screening and referral of chronic patients to clinics; and, referral of patients back to CHWs and THPs for monitoring in the community. The outputs required to attain the desired outcomes are trained health care providers, counselled community members, screened community members and referred patients. The outcome measures for these interventions would be improved quality of NCD management; increased access to NCD screening; increased knowledge about risk factors for NCDs; and, access to treatment and preventions strategies in the communities.

The intermediate expected outcomes as a result of the impact of the interventions will likely be a decrease in risk behaviour and an increase in access to NCD treatment. Decreased NCD risk factors, increased NCD incidences and decreased NCD morbidity and mortality will be assessed as the impact parameters of the interventions. The interventions in the proposed framework can strengthen the development of an integrated evidence-based chronic NCD management model, focusing on its implementation and later evaluation. This framework will be used to organise thinking around the model development; how to relate model activities and investment on expected results, which are improved chronic non-communicable disease management. The framework will also be used to set up performance indicators in the clinics; allocating responsibilities to all involved in chronic care and, finally, communicating information on the model implementation concisely and unambiguously.

### Inputs and activities to strengthen integrated evidence-based chronic non-communicable disease management model at Dikgale HDSS

**Fig. 2:**
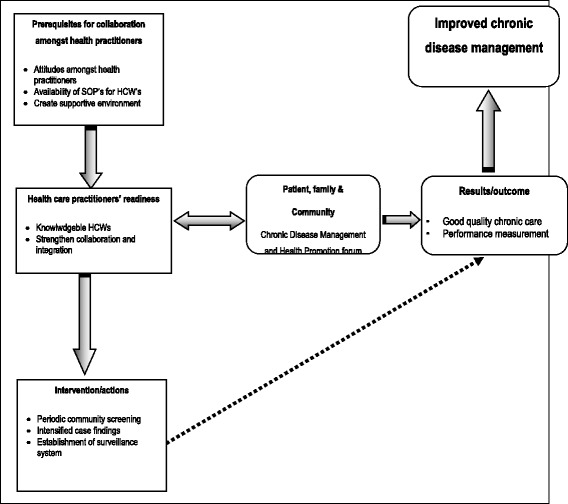
Prerequisites needed to strengthen integrated evidence-based chronic non-communicable disease management Dikgale HDSS

The discussions emanating from the QCs and executive management of the Limpopo Department of Health, together with Belgian Vlaamse Interuniversitaire Raad (VLIR) team, resulted in inputs and activities which are needed for the development and implementation of the interventions for improving CDM. Looking at the current health system in the Limpopo Province, the team suggested that the interventions should optimally and equitably utilise the current available healthcare resources to implement an integrated model which is culturally sensitive and appropriate to the population of the Dikgale area. The inputs and activities agreed upon to guide in the process to achieve improved chronic NCD management are outlined schematically in Fig. 2 as follows:

- There are prerequisites which were identified from the qualitative results that can be implemented to enhance collaboration among health care practitioners, such as addressing their attitudes. This can nurture the working relationship among nurses, CHWs and THPs. Again the provision or availability of Standard Operation Procedures (SOPs) for health care practitioners was emphasised in order to standardise quality health care services by nurses, CHWs and THPs. The creation of a supportive environment by the district and provincial offices for the health care workers was identified as a need to enhance the environment for all employees to improve their productivity and morale.
- Health care practitioners’ readiness can be improved by conducting training, which can impart knowledge to them and prepare them. Therefore, the strengthening of collaboration and integration of health care practitioner-services to serve the poor communities in the Dikgale area should be emphasised.
- The activities addressing periodic community screening targeting high risk groups and the raising of awareness on common NCDs and their risk factors in the community were identified. The findings from the qualitative study show that awareness campaigns should use approaches, such as community radio stations, community dialogue and mass campaigns, to reach more community members. Establishment of a surveillance system to monitor the occurrence of NCDs and their risk factors was also identified as an intervention needed in the health facilities.
- The involvement of the people themselves, family and community members, was seen as a critical aspect which can be done through the establishment of a chronic NCD management and health promotion forum. This forum should collaborate with community members to establish the chronic disease ambassador programme to will motivate patients in the communities through their health problems.

### Integrated evidence-based chronic non-communicable disease management model in Dikgale HDSS

**Fig. 3:**
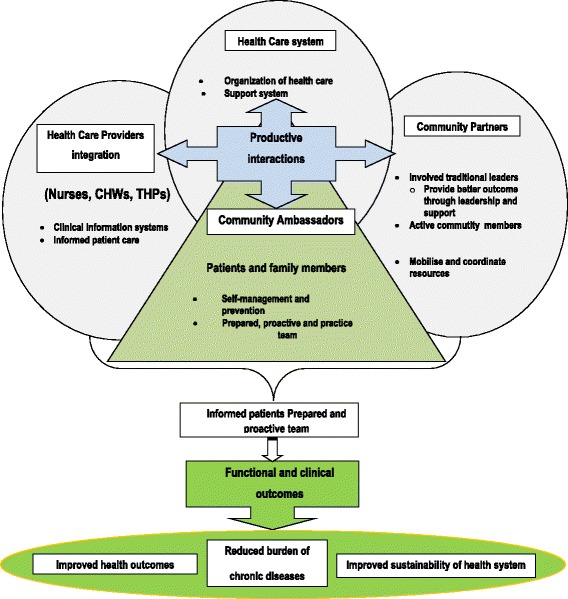
Integrated, evidence-based chronic non-communicable disease management model in the Dikgale HDSS

The logical framework (Fig. 1), together with the activities and inputs (Fig. 2), presented above led to the development of an integrated chronic non-communicable disease management model, which was the main objective of this study, and is presented in Fig. 3. As this model was developed and proposed based on the findings from the study, it is therefore evidence-based, i.e. based on the local situation in the Dikgale HDSS. It was developed in an integrated or coordinated approach to service delivery. This model describes four interacting system components, namely, health care providers, the health care system, community partners and patients with their families.

The main feature of this model is the integration of services provided by nurses, CHWs and THPs. A well-established clinical information system is proposed in the model for health care providers to have access to more informed patient care. The health care system based on this model will be tasked to organise health care in the rural area to improve management and prevention of chronic illnesses. Support systems in a form of supervisory visits to clinics, provision of medical equipment and the training of health care providers should be provided. A contribution from community partners, in the form of better leadership to mobilise and coordinate resources for chronic care, is emphasised in the model. This productive interaction will be supported by the district and provincial Health Departments through re-organisation of health services to give traditional leaders a role to participate in leadership and to improve community participation.

This study suggests patients may be used as ambassadors, informing others and will play a role to motivate other patients in the communities through their health problems. As the ambassadors will be drawn from those community members who are suffering from chronic diseases themselves but have managed their chronic illness well in the past, it will be good for chronic patients to learn from the best practices of those who managed their conditions well in the past. They will be tasked to encourage chronic patients on the self-management of their condition, together with the involvement of their family members and community partners at large. Informed patients who will form part of the prepared and proactive ambassador team will contribute to functional and clinical outcomes in the community. This will eventually lead to improved health outcomes, reduced burden of chronic diseases and improved sustainability of health system.

## Discussion

The integrated evidence-based chronic NCD management model developed in the current study describes changes which are needed at PHC settings in a rural area.

The main focus of our study was to develop appropriately effective intervention strategies that will facilitate change, in order to prevent and control chronic diseases within and across cultural contexts [28] in rural areas. An integrated multi-faceted approach to improving care of chronic conditions has been shown to result in better outcomes [32].

These changes are based on four interacting system components, which are health care providers; health care system; community partners; and, patients with their families. These health system changes are supported by the concept of an ideal clinic introduced into the South African health system, in order to move from a hospital-centric, curative system to preventive and promotive PHC that is cost effective and meets community needs [33]. This is also supported by the Chronic Care model developed by Wagner and colleagues, which comprises four components, which are self-management support; delivery system design; decision support; and clinical information systems [1, 34]. An important difference in the current proposed integrated model is the integration of services from nurses, CHWs and THPs. This addition to the model was mainly due to the fact that, in this rural area, health services are provided by nurses in the clinics and CHWs and THPs in the community. This study also supports the notion that patients may be the ambassadors who inform and support others, as seen in recent smoking cessation programmes [35] and also in decision making [36].

### Implications for health care and further research

There are two gaps which must be closed to achieve proper control of chronic diseases, which are the gap between effective interventions in research studies and what clinicians do in practice, and the gap between what clinicians in their offices recommend to patients and what patients do at home and in their communities [37]. To successfully close these gaps, we have discussed the interventions with experts in CDM, including clinicians and researchers, together with the executive management of the Department of Health in Limpopo Province, as well as evidence collected from the health facilities in the study area. The public health implications from the study are that our interventions are oriented towards health promotion and prevention through a primary health care approach in order to effectively respond to the complex social, cultural and behavioural issues associated with NCDs.

## Conclusions

The model developed highlights the need for health interventions that aim to control risk factors at the population level, the need for availability of NCD-trained nurses, functional equipment and medication and a need to improve the link with traditional healers. Therefore, further research will be conducted in the study area on planned community intervention programmes, which are a substantial component of the strategy to help solve prevention of NCDs [8]. Therefore, as the model development is comprehensive [37, 38] emphasis should also be placed on the complex and dynamic settings in which the delivery of nursing care occurs, as it involves social, political, economic and clinical factors [39].

## Abbreviations

CDM: Chronic disease management
CHWs: Community health workers
HDSS: Health and Demographic Surveillance System
MRC: Medical Research Council
NCD: Non-communicable disease
PHC: Primary health care
QCs: Quality circles
QI: Quality improvement
THPs: Traditional health practitioners
VLIR: Vlaamse Interuniversitaire Raad
WHO: World Health Organization
